# The expression pattern of immune-related genes and characterization of tumor immune microenvironment: predicting prognosis and immunotherapeutic effects in cutaneous melanoma

**DOI:** 10.1186/s12957-022-02767-z

**Published:** 2022-09-22

**Authors:** Dong Dong, Wei Wang, Heng Wang, Liang Chen, Tianyi Liu

**Affiliations:** Department of Plastic and Aesthetic Surgery, Huadong Hospital, Fudan University, Shanghai, 200040 China

**Keywords:** Immune, Melanoma, Tumor immune microenvironment, Immunotherapy, TCGA

## Abstract

**Background:**

Increasing evidences have revealed the tumor immune microenvironment not only has vital impacts on the origin, progression, and metastasis of tumors significantly but also influences the response to immunotherapy. Nonetheless, to date, the well-rounded expression pattern of immune-related genes in cutaneous melanoma and the comprehensive characterization of tumor immune microenvironment remain not clearly elucidated.

**Method:**

We comprehensively evaluated the well-rounded expression pattern of immune-related genes of 686 patients with cutaneous melanoma based on immune-related genes with prognostic value and systematically correlated the expression pattern of these genes with the comprehensive characterization of tumor immune microenvironment. The IRGscore was constructed to quantify immunological function of individual using principal component analysis algorithms.

**Result:**

Three distinct immune subtypes were determined with obvious survival differences. Melanoma patients with high IRGscore was characterized by comprehensive suppression of immune function, showing much poorer prognosis and efficacy for immunotherapy, while the low IRGscore means the robust activation of immune function and the better effect of immunotherapy, which may be responsible for a better prognosis. Besides, the prognostic ability of IRGscore was further validated by the independent dataset of stomach cancers. Furthermore, the predictive effect of immunotherapeutic benefits of IRGscore was demonstrated by the independent dataset of melanoma patients accepting immunotherapy and another predictive model for immunotherapy.

**Conclusion:**

IRGscore could serve as an independent immunotherapeutic and prognostic predictor, thereby facilitating the identification of appropriate candidates with cutaneous melanoma for immunotherapy and the formulation of individualized therapeutic approaches.

**Supplementary Information:**

The online version contains supplementary material available at 10.1186/s12957-022-02767-z.

## Introduction

Cutaneous melanoma (CM) is one of the most aggressive malignant skin tumors characterized by metastasis at an early stage and poor prognosis [1]. Epidemiological evidences indicate the incidence of CM has increased drastically worldwide in the past few years, contributing to 80% of deaths from dermatologic cancers [2, 3]. It causes approximately 55,500 deaths annually, and less than 20% of individuals with advanced CM could survive 5 years [4].

Over the past 10 years, the therapeutic strategies for advanced CM have progressed dramatically with the development of immunotherapy represented by immunological checkpoint blockade (ICB), which could effectively promote the activation of the immune system and improve anti-tumor immune response [5]. Nevertheless, an apparent restriction of ICB, as observed, is merely a tiny percentage of CM patients with durable responses could benefit from it, whereas there is no objective response for 60–70% of CM patients to immunotherapy, and 20–30% of these patients without objective response end up with tumor recurrence and progression [5–7]. Therefore, reliable indicators or predictors are extremely in demand to help identify the appropriate CM individuals for immunotherapy.

Increasing evidences have revealed the strong correlation between the response of patients to immunotherapy and the immune composition of tumor microenvironment (TME) [8]. The TME comprises a variety of immune cells together with endothelial cells, fibroblasts, and extracellular components, which not only has vital impacts on the origin, progression, and metastasis of tumors significantly but also influences the response to immunotherapy [9]. According to previous researches, the high level of tumor-infiltrating lymphocytes (TIL), such as NK cells, CD8+ T cells, CD4+ T cells, and activated B cells, was generally relevant to durable response to immunotherapy and better prognosis [10, 11]. Activated CD8+ T cells could directly recognize and kill malignant tumor cells. And CD4+ T cells have been demonstrated to improve the effectiveness of CD8+ T response and secrete various cytokines to promote immune response. Furthermore, CD4+ T cells, via cytolytic mechanisms, are also capable of destroying tumor cells directly [12]. However, simply evaluating TIL cannot absolutely predict the response to immunotherapy, and some individuals with high levels of TIL were also observed the resistance to immunotherapy [13, 14]. This could be explained by the fact that the response to immunotherapy is also impacted by various cytokines, chemokines, and other components of tumor immune microenvironment (TIM) [15].

More specifically, infiltrating immune cells of tumor are heterogeneous in both function and phenotype and make up an interactive network with other immune cells and components of TIM, thereby constituting an extremely complex integration [16, 17]. For example, CD4 + FOXP3 + regulatory T cells (Treg) have crucial effects on the establishment and preservation of self-tolerance [18]. Nevertheless, as suppressors of immune responses, the infiltrating level of Treg in tumors is generally associated with a poor prognosis [19]. Through humoral and cell-cell contact mechanisms, Tregs could suppress not only T cells but also NK cells, macrophages, dendritic cells, and B cells [20]. Besides, it has been well demonstrated tumor-associated macrophages (TAMs) are also the key regulators of immune response, which are capable of excreting many suppressive cytokines including IL-1β, TGFβ, IL-10, and IL-6, thereby leading to the suppression of T cell in the TIM [21, 22]. Therefore, more attention should be paid to the interaction among various components of TIM, rather than a single-cell cluster. The comprehensive characterization of the TIM and the expression pattern of immune-related gene might be a valuable reference in formulating individualized treatment strategies.

The present work depicted the comprehensive landscape of TIM in CM, revealing that characterization of TIM and the immune-related gene expression pattern of individuals were closely correlated to tumor heterogeneity and treatment complexity. Furthermore, a reliable scoring system has been established in this study, serving as an independent immunotherapeutic and prognostic indicator, to quantify immune status of individual tumors and comprehensively evaluate the response of CM patients to immunotherapy, thereby assisting the formulation of individualized therapeutic strategies.

## Material and methods

### CM dataset acquisition and preprocessing

The detailed workflow of this research was depicted in Supplementary Fig. 1. Firstly, we searched and downloaded the public gene-expression data as well as complete clinical annotation from The Cancer Genome Atlas (TCGA) and Gene Expression Omnibus (GEO) database. Those individuals with full survival information were screened for subsequent analysis. In this study, 685 CM samples datasets (TCGA-SKCM and GSE65904) were identified altogether for further evaluation. As to datasets in TCGA, RNA-sequencing data of gene expression (FPKM values) were obtained from the University of California Santa Cruz (UCSC) Xena Browser (Genomic Data Commons [GDC] hub: https://xenabrowser.net/datapages/?hub=https://gdc.xenahubs Accessed September 15, 2021). For datasets in GEO, we directly obtained the matrix files after normalization. More specifically, we converted the FPKM values into transcripts per kilobase million (TPM) values. Using the “ComBat” algorithm of sva package, we corrected the batch effects from non-biological technical biases were corrected. Besides, we downloaded the somatic mutation data from TCGA database. R Bioconductor and R (version 4.1.1) packages were employed for performing data analysis. In addition, the independent CM datasets (GSE19234) were analyzed to validate the predictive ability of IRGscore to prognosis. Besides, the independent CM datasets (GSE91061), including 49 CM individuals receiving immunotherapy, were analyzed to explore the predictive ability of IRGscore to immunotherapy.

### Unsupervised clustering for immune-related genes with a prognostic ability

We identified a gene set including 6196 genes related to immune function through the Immunology Database and Analysis Portal database [23]. The prognostic values of these immune-related genes in CM patients were revealed by the univariate Cox regression model. Then, 742 genes related to immune function with prognostic value (*P* < 0.05) were screened from the results file for subsequent analysis. Based on the expression of 742 genes related to immune function with prognostic value, we utilized unsupervised clustering analysis to determine different immune subtypes and divide CM individuals for subsequent analysis. The consensus clustering algorithm determined the quantity and stability of clusters. The ConsensuClusterPlus package [24] was used to carry out the previous processes, and 1000 times repetitions were performed to guarantee the stability of categorization.

### Identification of differentially expressed genes (DEGs) among distinct immune subtypes

Based on selected genes related to immune function with prognostic value, individuals with CM were divided into three distinct immune clusters. The empirical Bayesian approach of limma R package was utilized to identify DEGs among distinct immune clusters. In addition, the significance filtering criteria of identifying DEGs were set as an adjusted *p*-value < 0.001.

### Estimation of immune cell infiltration of TME by single-sample gene set enrichment analysis (ssGSEA) and CIBERSORT deconvolution algorithm

We used the R package “CIBERSORT” to quantify the infiltrating level of various immune cells in melanoma for 1000 permutations. Besides, the stromal/immune cells (stromal/IRGscores) were assessed by ESTIMATE algorithm [25]. Then, the standard scores were calculated, and the calculation formula of standard score is “*Z* = (X–X _bar)/S.” X is the original score, X_bar is the mean of the original score, and S is the standard deviation of the original score. Additionally, the relative abundances of immune cells within TME were quantified by ssGSEA algorithm. And the gene panels used to mark diverse immune cell types of TIM were acquired from an article of Charoentong [26]. The relative abundance of each immune cell type in TIM was denoted by an enrichment score determined by ssGSEA analysis.

### Gene set variation analysis (GSVA) and functional annotation

Based on the R package “GSVA” function, the distinctions in biological processes among three immune clusters were further evaluated by GSVA enrichment analysis, which was an unsupervised and nonparametric approach for assessing the variations in signaling pathways and the activity of biological process in the samples [27]. The adjusted *P* < 0.05 was considered statistically significant. Using the clusterProfiler R package, the Kyoto Encyclopedia of Genes and Genomes (KEGG) and gene ontology (GO) functional annotation for DEGs were carried out, with a cutoff value of false discovery rate (FDR) < 0.05.

### Generation of IRGscore

The DEGs determined from distinct immune clusters were firstly normalized among all samples, and the overlapping DEGs were selected. Using unsupervised clustering method, individuals were categorized into different subtypes for sequent analysis based on the overlapping DEGs. Then, we utilized the consensus clustering algorithm to identify the quantity and stability of three gene clusters. Furthermore, via univariate Cox regression model, the prognostic analysis for each gene in the signature was carried out, and we extracted those genes with the prominent prognostic value for further analysis. Then, principal component analysis (PCA) was performed to establish the gene signatures related to immune, with principal components 1 and 2 being the signature scores. The advantage of this approach is to focus the score on the set containing significantly well-correlated or anti-correlated genes whereas down-weighing the contribution from genes that do not track with other members of the set. The approach of defining the IRGscore in our study is similar to GGI [28]:

$$\mathrm{IRGscore}=\sum \left(\mathrm{PC}1\mathrm{i}+\mathrm{PC}2\mathrm{i}\right)$$

where *i* represented the expression of immune-related genes.

### Quantify the immune response predictor: immunophenoscore (IPS)

IPS is the most favored factor, developed by Charoentong P. et al., to predict the anti-PD-1 and anti-CTLA-4 responses, quantifying immunogenicity determinants of tumors, and characterizes immune landscapes within the tumor as well as cancer anti-genome [29]. The ESTIMATE algorithm, using the distinct transcriptional patterns for inferring tumor purity and cellularity, was utilized to determine stromal/immune scores for predicting stromal/immune cell infiltrating degrees [30]. Tumor tissues which had plentiful immune cell infiltration meant lower tumor purity and a higher IPS.

### Statistical analysis

All statistical analyses in this study were finished in R 4.1.1 software. Correlation coefficients between two variables were calculated by Spearman’s and distance correlation analyses. Kruskal-Wallis tests and one-way ANOVA were utilized to contrast differences across three subgroups [31]. The optimal cutoff point of each group, using the survminer R package, was identified based on the correlation between IRGscore and patients’ survival. Besides, we utilized the surv-cutpoint function of the “survival” package to tautologically examined all possible cut points for identifying the maximum rank statistic, used to dichotomize IRGscore, and divided individuals with CM into low and high IRGscore groups. By the Kaplan-Meier method, we depicted the survival curves for prognostic analysis, and the significance of variations was examined by log-rank tests. Furthermore, we used the multivariable Cox regression model to ascertain independent prognostic factors. Patients who had full clinical information were selected to perform further multivariate prognostic analysis. In addition, we utilized the forestplot R package for visualizing the data of multivariate prognostic analysis for IRGscore in CM cohort. All statistical *P*-values were bilateral, and *P* less than 0.05 was deemed as statistically significance.

## Results

### Identification of immune subtypes

We identified a gene set including 6196 genes related to immune function through the Immunology Database and Analysis Portal database [23]. Based on 686 tumor samples with available clinical information and OS data profiles from the meta-cohort (GSE65904; The Cancer Genome Atlas [TCGA]-SKCM), a univariate Cox regression model revealed the prognostic values of the above immune-related genes in patients with CM. Then, 742 genes related to immune function with prognostic value (*P* < 0.05) were screened from the results file for subsequent analysis.

**Fig. 1:**
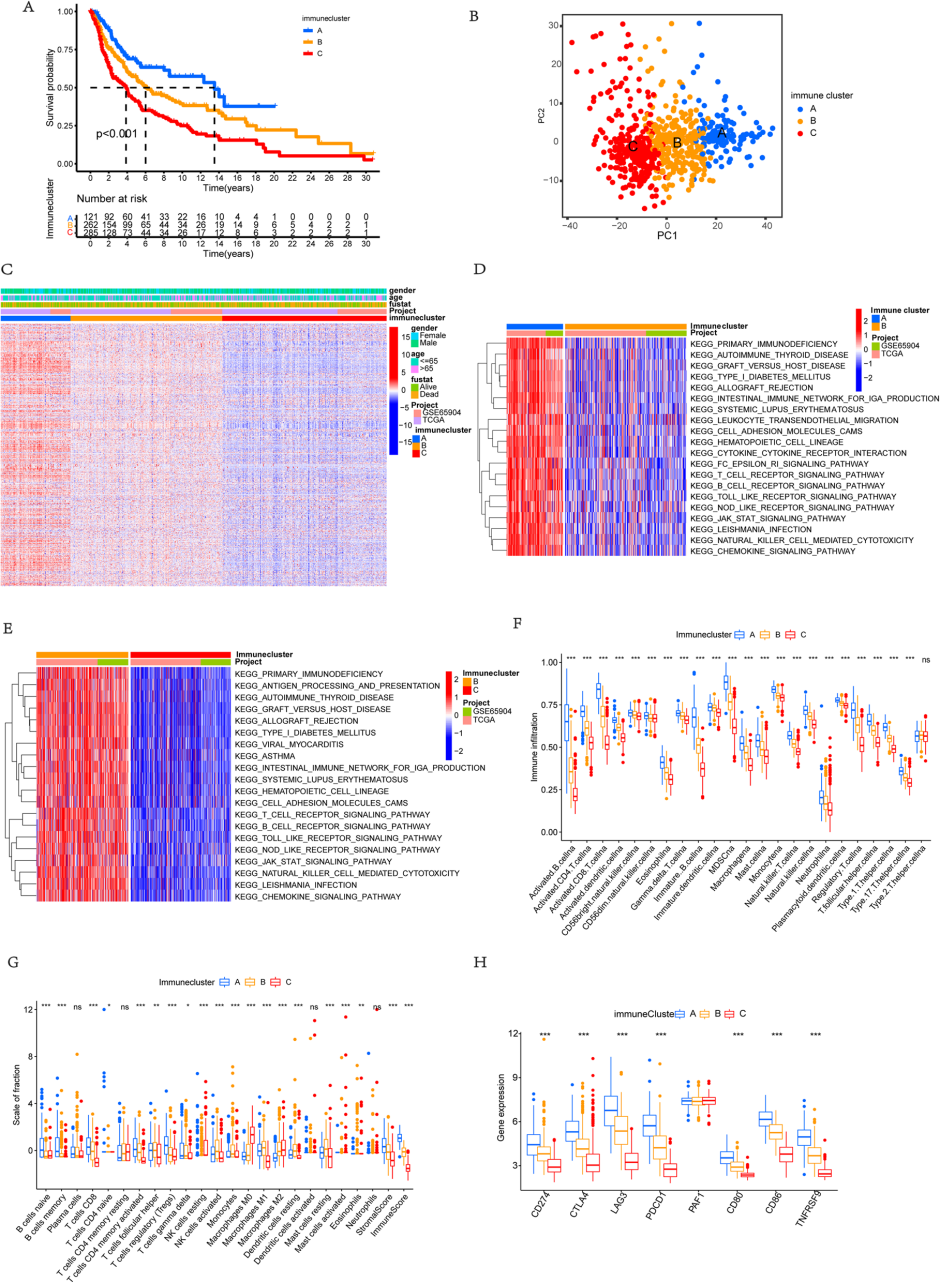
Immune landscape and functional annotation of different immune subtypes. **a** Survival analysis of three immune clusters in the combined CM cohort. K-M curves with *P* < 0.001 suggested that the difference in survival was significant across the three clusters. Immune cluster A displayed superior survival compared with the other clusters. **b** PCA for the transcriptome profiles of the three immune clusters, suggesting an obvious distinction in the transcriptome of among different subtypes. **c** Unsupervised clustering of the genes related to immune in the combined CM cohort. Immune cluster, survival status, and age served as patients’ annotations. Red means high expression levels, and blue represents low expression levels of these genes. **d** and **e** GSVA enrichment analysis of the activated signaling pathways in three different immune clusters. Red color means the activation of signaling pathways, and blue represents the inhibition of signaling pathways. **f** Variations in the abundance of infiltrating immune cells among immune clusters A, B, and C using “ssGSEA” analysis. “*” represents the statistical *P*-value (*P < 0.05; **P < 0.01; ***P < 0.001). **g** Difference in the abundance of infiltrating immune cells among immune clusters A, B, and C using “CIBERSORT” analysis. The value of Y-axis is standard score. “*” represents the statistical *P*-value (*P < 0.05; **P < 0.01; ***P < 0.001). **h** The expression of immune-checkpoint-relevant genes expressed in three immune clusters (*P < 0.05; **P < 0.01; ***P < 0.001)

Using the ConsesusClusterPlus package of R software, the unsupervised clustering was carried out to divide patients with CM into separated subtypes based on the expression level of these 742 genes related to immune. Three independent immune subtypes were identified with significant survival differences. Immune cluster A showed a particularly noticeable survival advantage among three distinct immune clusters, according to prognostic analysis, while the immune cluster C had a worst prognosis (Fig. 1a). Additionally, PCA has demonstrated the obvious differences among three different immune clusters about the transcriptional profile of these 742 genes related to immune (Fig. 1b). According to heat map analysis, the expression levels of these genes in immune cluster A patients were significantly higher than those in immune cluster B and cluster C patients (Fig. 1c).

### Immune landscape and functional annotation of different immune subtypes

To probe into the biological characteristics among three different immune clusters, we performed the GSVA enrichment analysis. Immune cluster A presented remarkable enrichment of signaling pathways related to activation of immune, including toll-like receptor signaling pathways, T-cell receptor signaling pathway, B-cell receptor signaling pathway, chemokine signaling pathway, and the cytokine-cytokine receptor interaction signaling pathway, while the immune cluster C was significantly associated with the biological process of immune suppression. And the level of immune activation of immune cluster B lied between immune cluster A and immune cluster C, all of which were consistent with the results of prognostic analysis (Fig. 1 d and e). To further clarify the differences in immune function among distinct immune subtypes, the components of immune cells in the immune microenvironment were analyzed. The ssGSEA results, as expected, revealed various infiltrating immune cells were observed to be prominently enriched in immune cluster A, including CD4+ T cells, gamma-delta T cells, macrophages, activated B cells, mast cell, MDSC, natural killer cell, CD8+ T cells, and eosinophils (Fig. 1f). While the immunecluster-C was characterized by the comprehensive immune suppression. We further assessed the proportion of different subtypes of infiltrating immune cells of tumors based on the “CIBERSORT” method (Fig. 1g). Results also indicated higher levels of immune effector cells in immune cluster A, such as activated CD4+/CD8+ T cells and memory CD4+/CD8+ T cells, which was consistent with the above results of ssGSEA. Besides, the expression levels of several vital immune checkpoint genes were also analyzed, including CTLA4, PD-L1, LAG3, PAF1, PD1, CD80, CD86, and TNFRSF9 in each immune clusters. It has been demonstrated that the above expressions of immune checkpoints except PAF1 in immune cluster A were significantly higher than that in immune cluster B, and the cluster C was still characterized by the lowest expression level of these immune checkpoints among immune subtypes (Fig. 1h).

### Construction of immune-related gene signatures and identification of immune gene subtypes

**Fig. 2:**
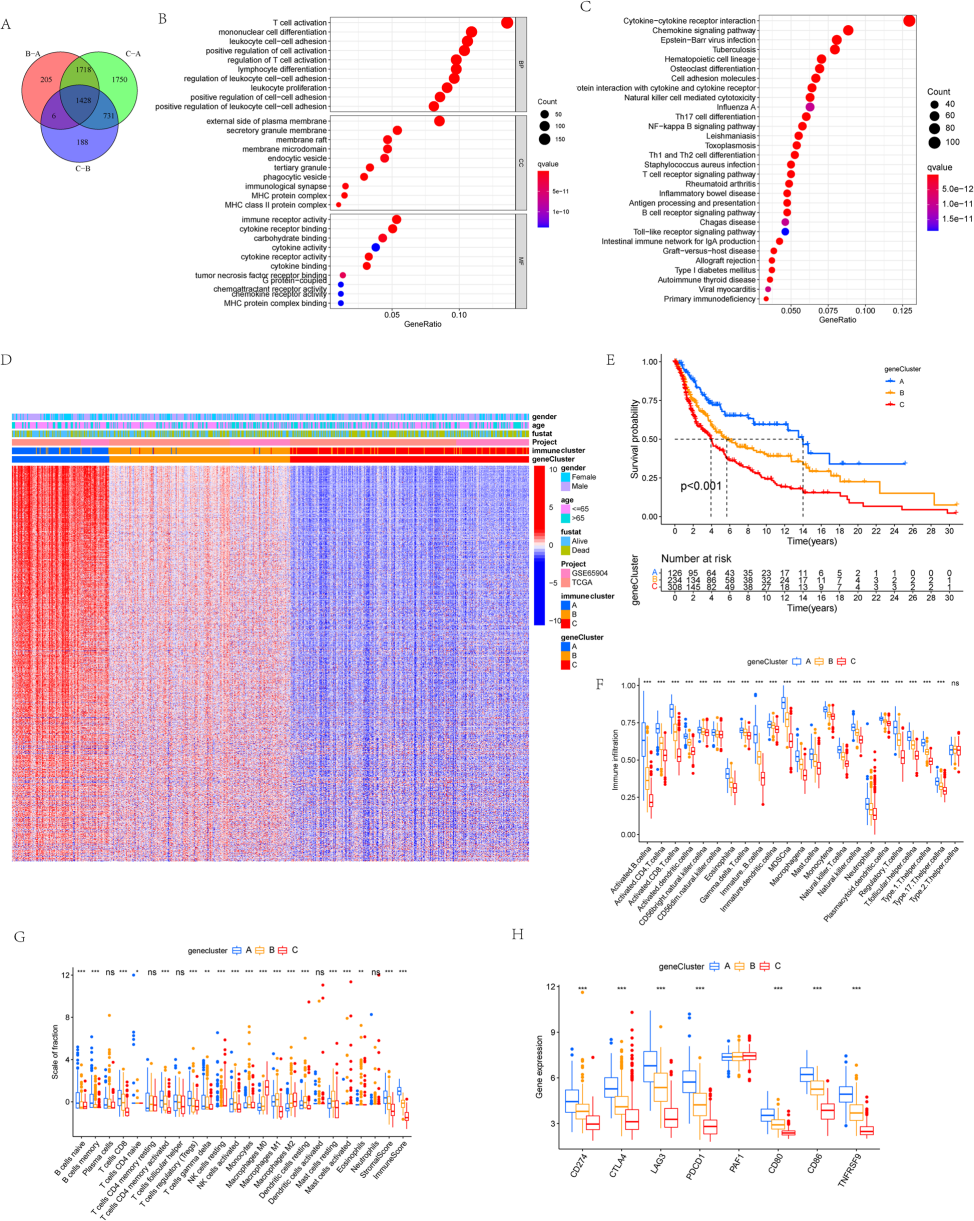
Construction of immune-related gene signatures and identification of immune gene subtypes. **a** Venn diagram presented 1428 overlapping DEGs among three immune clusters was identified. **b** Functional annotations of DEGs based on GO analysis and the circle size mean the number of genes enriched. **c** Functional annotations of DEGs based on KEGG pathway analysis and the circle size represent the enriched gene number. **d** Unsupervised clustering of the immune-related gene signatures in the combined CM cohort. The immune cluster, gene cluster, survival status, and ages served as patient annotations. Red means high expression levels, and blue represents low expression levels of these genes. **e** Survival analysis of distinct gene clusters in the combined CM cohort. K-M curves with *P* < 0.001 suggested that the difference in survival was significant across the three clusters. **f** Variations in the abundance of infiltrating immune cells across gene clusters A, B, and C using “CIBERSORT” analysis. The value of Y-axis is standard score. “*” represents the statistical *P*-value (*P < 0.05; **P < 0.01; ***P < 0.001). **g** Differences in the abundance of infiltrating immune cells among gene clusters A, B, and C using “ssGSEA” analysis. “*” represents the statistical *P*-value (*P < 0.05; **P < 0.01; ***P < 0.001). **h** The expression of genes related to immune checkpoints in three gene clusters

To further unravel the potential biological characteristics of each immune subtypes, using the limma packages of R software, the differential analyses of gene expression among three immune clusters were performed to identify the transcriptome distinctions, finally determining 1428 immune subtype-related overlapping differentially expressed genes (DEGs) (Fig. 2a). Then, we utilized the clusterProfiler package to carried out GO and KEGG enrichment analysis for these DEGs (Fig. 2 b and c). And as expected, these genes were prominently enriched in biological processes associated with immune function, involving T-cell activation, leukocyte proliferation, lymphocyte differentiation, and mononuclear cell differentiation, which confirmed again that the expression pattern of immune-related gene played a vital role in the survival differences among three immune clusters. Next, the above overlapping DEGs were utilized for performing survival analysis for each gene by the univariate Cox regression model, and final 1161 most prognostic DEGs were identified, together constituting the immune-related gene signatures. And the heat map delineated the transcriptomic profile of these prognostic DEGs with identified across the immune clusters (Fig. 2d). For better validating the above regulatory mechanism, we performed the unsupervised clustering of these immune signature genes detected in three immune clusters, which classified the GSE65904 and TCGA-SKCM cohort into distinct gene subtypes. Consistent with the immune subtypes, three distinct genomic phenotypes were recognized via an unsupervised clustering algorithm, termed as gene cluster A, gene cluster B, and gene cluster C, respectively.

The prognostic characteristics of three gene clusters were investigated by integrating them with survival information. One-hundred twenty-six of 300 patients with CM were aggregated in gene cluster A, suggesting a better survival, while patients in gene cluster C (308 patients) were observed to be strongly associated with poorer outcomes. Besides, 234 patients with CM belongs to gene cluster B with an intermediate prognosis (Fig. 2e). Furthermore, it has been investigated that the landscape of immune cell infiltration in the TIM in three gene clusters based on “CIBERSORT” and “ssGSEA” methods (Fig. 2 f and g). We found the gene cluster A had dramatically higher IRGscores compared with other gene clusters, and it exhibited the highest activated CD8^+^ T cell and CD4^+^ memory T-cell infiltration. As depicted in Fig. 2f, gene cluster C, with much lower IRGscores, was characterized as the remarkable immunosuppression-related M2 macrophages infiltration. Additionally, to further explore the biological behaviors among three gene clusters, we also explored the expression levels of some vital immune checkpoint genes in three gene clusters, indicating significant differences. The gene cluster A was related to much higher expression levels of immune checkpoints, whereas the lowest gene expression level was observed in gene cluster C (Fig. 2g). In brief, the coherence between prognostic profile and immune profile among distinct gene clusters has indicated the sorting scheme is reasonable and scientific.

### Establishment of the immune-related gene score (IRGscore)

**Fig. 3:**
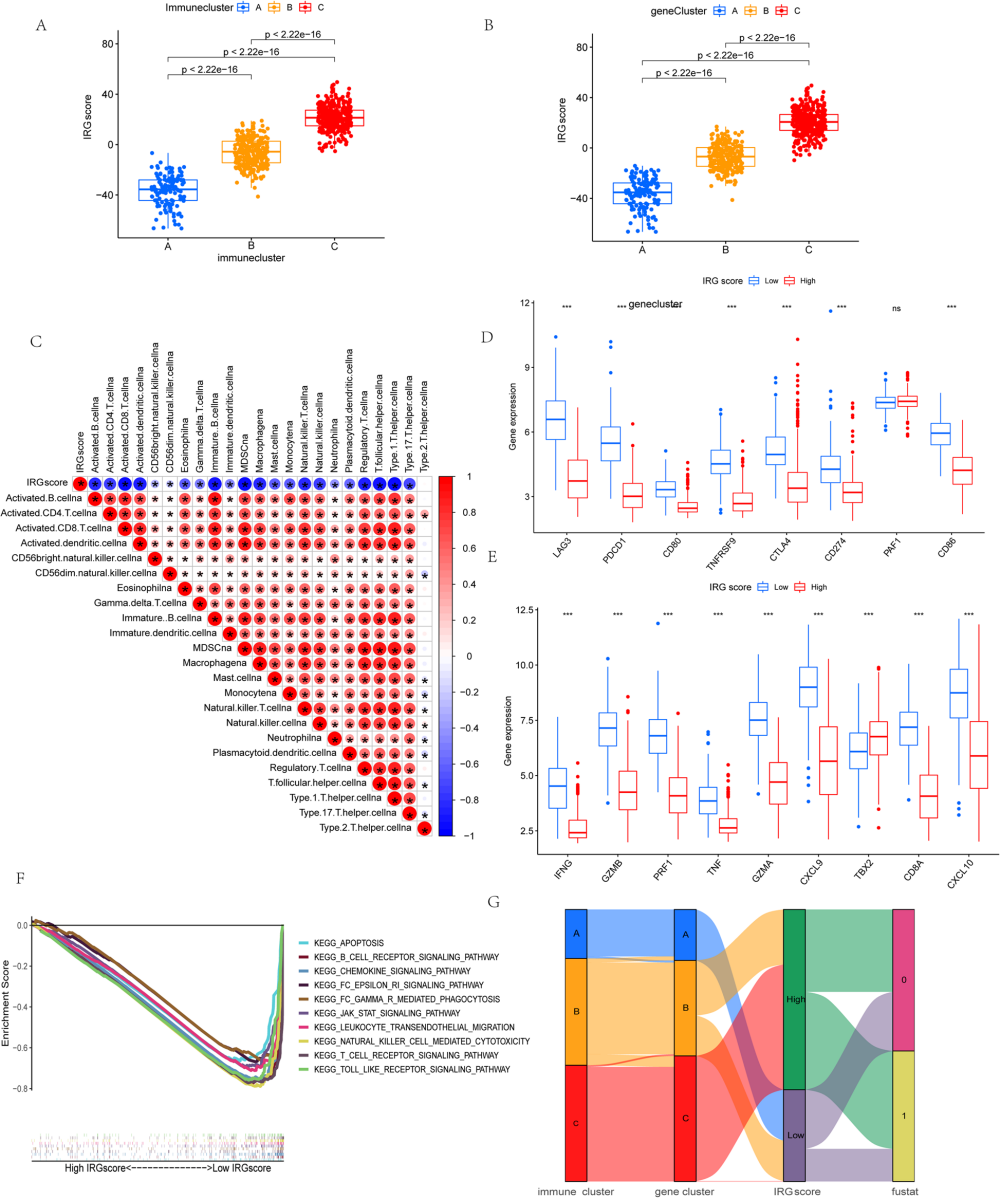
Construction of the IRGscore. **a** IRGscore differences among three immune clusters (*P* < 0.001, Student’s *t*-test). **b** Differences in IRGscore across three gene clusters (*P* < 0.001, Student’s *t*-test). **c** Correlations between IRGscore and immune infiltrating cells of TME analyzed by Spearman’s analysis. Red and blue colors represent positive and negative correlations, respectively. **d** The expression of genes related to immune checkpoints in low and high IRGscore groups. **e** The expression of immune activation-related genes in high and low IRGscore groups. **f** GSVA analysis (c2.cp.kegg.v7.4.symbols.gmt) revealed that the signaling pathways related to immune activation were remarkably enriched in low IRGscore samples. **g** The alluvial diagram indicated the attribute alterations in different patterns

To acquire quantitative predictors of immune function in CM individual patients, based on the above immune-related gene signatures, we constructed a set of scoring system to quantify immune-related gene expression pattern of individual patients with CM, termed as IRGscore. Detailed constructive processes of IRGscore are provided in the chapter of methods. Additionally, the Kruskal-Wallis test further revealed remarkable differences in IRGscore among distinct gene clusters and immune clusters (Fig. 3 a and b). The lowest average score was attached to gene cluster A, whereas the gene cluster C was along with the highest average score among three clusters, suggesting that IRGscore was negatively correlated with immune function, and high score meant immune suppression, while a low score might be related to immune activation. Additionally, further analysis also indicated the IRGscore was significantly negatively associated with the level of infiltration of various immune cells including activated B cells, CD8 T cells, and CD4 T cells (Fig. 3c). To further verify this characteristic, we classified individuals with CM into low or high IRGscore group with the optimum cutoff value determined by survminer package; the tolerance condition and immune activity were further analyzed in low/high IRGscore groups. Firstly, we analyzed the expression level of several vital immune checkpoint genes, such as CTLA4, PAF1, CD80, PD-L1, LAG3, CD86, PD1, and TNFRSF9, as well as the expression level of signatures related to immune activity, such as PRF1, TNF, CXCL9, GZMB, GZMA, IFNG, CXCL10, TBX2, and CD8A. The Wilcoxon test has indicated that most of key genes related to immune activity and immune checkpoints were substantially upregulated in low score group, except PD2 and TBX2. (Fig. 3 d and e). Additionally, the gene set enrichment analysis (GSEA) also demonstrated that immune-related pathways were evidently elevated in low IRGscore group, such as toll-like receptor and T-cell receptor signaling pathways, B-cell receptor signaling pathways, and NK cell-mediated cytotoxicity pathways (Fig. 3f). The alluvial diagram indicated the attribute alterations in different patterns. As depicted, almost all individuals belonged to immune cluster C were also corresponding to gene cluster C, all of which had higher IRGscores and worse survivals. In contrast, most of patients, belonged to immune cluster A, were also corresponded to gene cluster A, meaning lower IRGscores and better outcomes (Fig. 3g).

### The prognostic ability of IRGscore

**Fig. 4:**
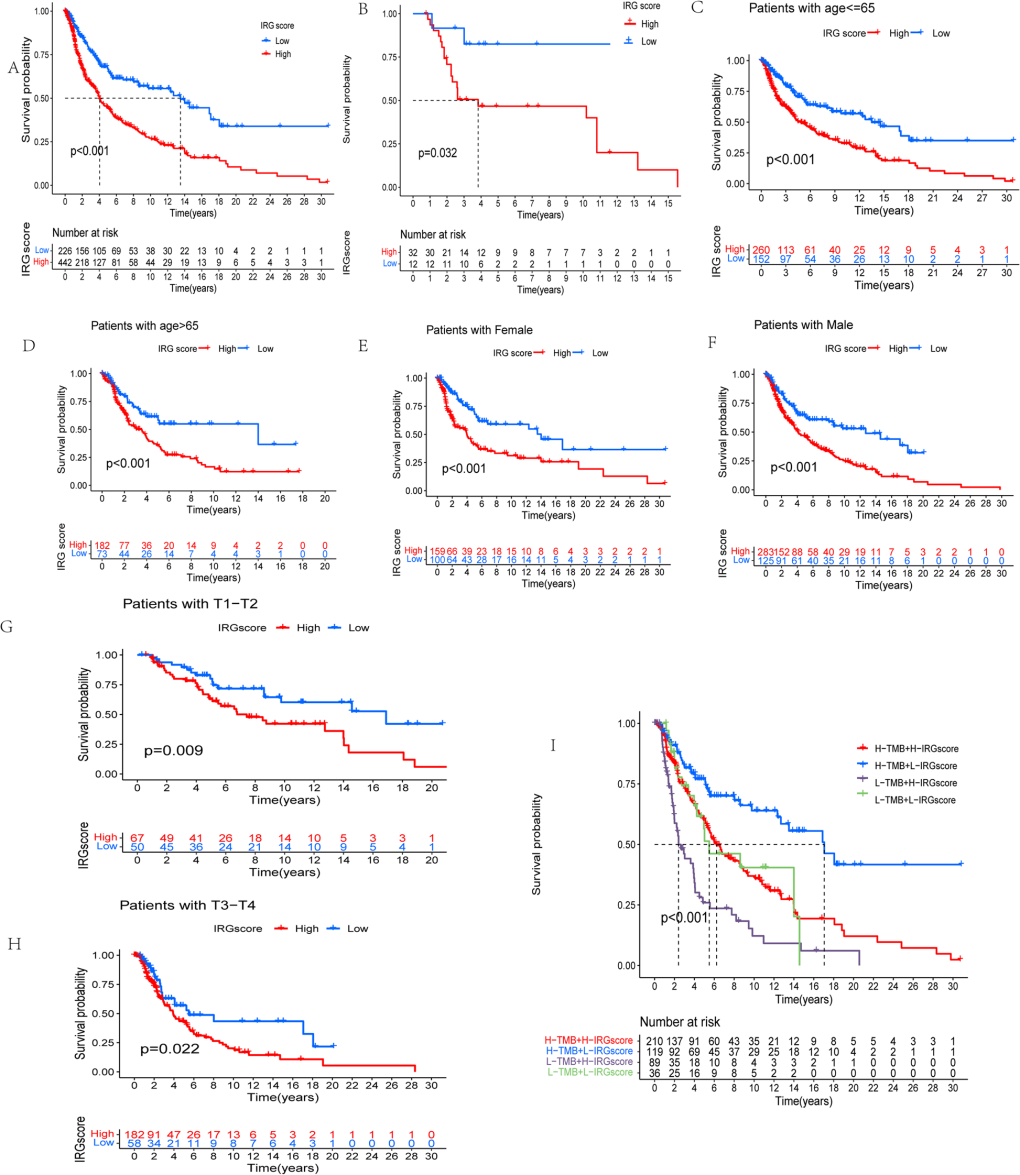
The prognostic ability of IRGscore. **a** Survival analysis of melanoma patients with low and high IRGscore based on K-M curves (*P* < 0.0001, log-rank test). **b** Survival analysis of independent dataset of cutaneous melanoma patients with low and high IRGscore based on K-M curves (*P* < 0.05, log-rank test). **c** Age < = 65 (*P* = 0.022, log-rank test). **d** Age > 65 (*P* = 0.037, log-rank test). **e** Female (*P* = 0.012, log-rank test). **f** Male (*P* = 0.014, log-rank test). **g** Patients with stage T1-2 (*P* = 0.081, log-rank test). **h** Patients with stage T3-4 (*P* = 0.045, log-rank test). **i** Stratified survival analysis of patients derived from TCGA-SKCM cohort divided according to both IRGscores and TMB (*P* < 0.001, log-rank test)

The subsequent analysis further evaluated the value of IRGscore in predicting patients’ outcome. Patients with low IRGscore had a significant survival advantage, compared with the high IRGscore group (*P* < 0.001) (Fig. 4a). Besides, the prognostic value of IRGscore was further validated based on an independent dataset of CM patients (GSE19234). As expected, the survival of patients belonging to low score group was also prominent better than high score group (*P* < 0.05) (Fig. 4b). Additionally, our present work also explored whether IRGscore was an independent predictor to evaluate the prognosis of patients with CM. Based on multivariate Cox regression model analysis, the predictive ability of IRGscore has been demonstrated to be independent of patients’ gender (*P* < 0.001), age (*P* < 0.001), and ACJJ T stage (*P* < 0.01), indicating this score system could exert its predictive effect as an independent, reliable, and effective biomarker (Fig. 4 c–h).

Numerous researches have demonstrated tumor burden mutation (TMB) might influence outcomes of the CM patients and the response to ICB [7, 32]. An increased TMB are always associated with a better immune-therapeutic effect and prolonged progressive-free survival [33]. Considering the prominent clinical implications of TMB, the functional relationships between the IRGscores and TMB were investigated to decipher the genetic signatures of distinct immune clusters. Firstly, based on the set point of TMB, patients with CM were divided into separate subtypes, we observed patients belonging to high TMB group indicated better survival than the individuals with the low TMB, as shown in Supplementary Fig. 2, which was consistent with previous researches [34]. Next, we compared the TMB of patients with low IRGscore and high IRGscore groups. Nevertheless, there was no any statistic differences of TMB between the low and high score group (Supplementary Fig. 2). Via stratified survival analysis, our present work further revealed that the predictions based on IRGscore were not disturbed by TMB status. Both in low and high TMB subgroups, the remarkable prognosis variations were observed between low/high IRGscore group (Fig. 4i). To sum up, these results further demonstrated the IRGscore was an independent predictor which could effectively evaluate the outcomes of patient with CM.

### The effects of IRGscore in assessing immunotherapeutic benefits

**Fig. 5:**
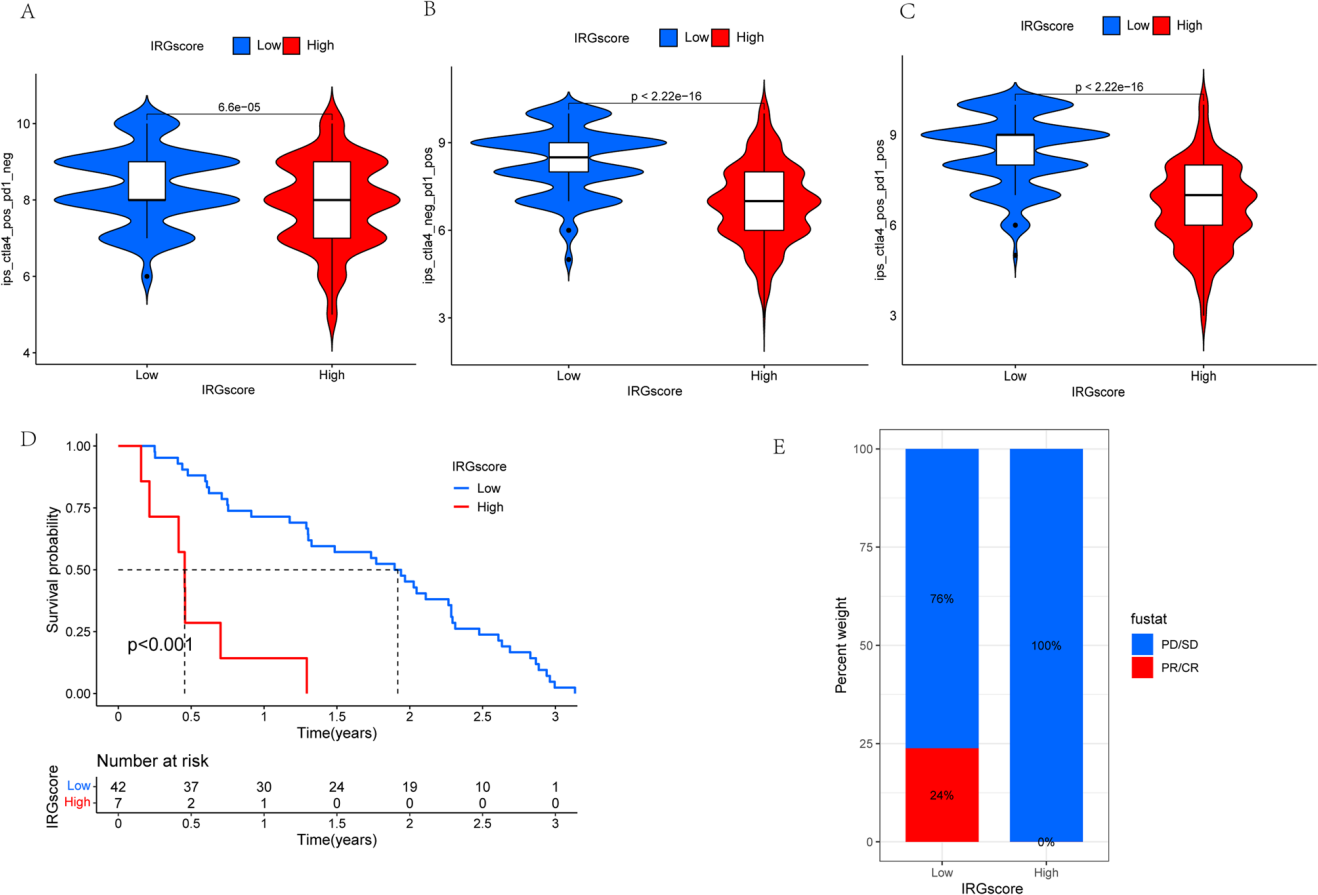
The role of IRGscores in the prediction of immunotherapeutic benefits. **a** The immunophenoscore of anti-CTLA-4 immune checkpoint therapy in melanoma patients with the low and high IRGscore. **b** The immunophenoscore of anti-PD-1 immune checkpoint therapy in melanoma patients with the low and high IRGscore. **c** The immunophenoscore of anti-PD-1 and CTLA-4 immune checkpoint therapy in melanoma patients with the low and high IRGscore. **d** Survival analysis of patients with low and high IRGscore from the cohort consisting of CM patients receiving immunotherapy (GSE91061) based on K-M curves (*P* < 0.0001, log-rank test). **e** Proportions of PD-1 blockade immunotherapy-responsive patients in high and low IRGscore groups. CR, PR, and PD stand for complete response, partial response, and progressive disease

ICB has brought revolutionary advances in the fields of cancer therapies, demonstrating an unprecedented increase of patient’s survival. Unfortunately, an obvious restriction of ICB is merely a minor percentage of CM patients with durable responses could benefit from it, whereas the majority experienced little clinical benefit. The effects of the IRGscore in evaluating the response to ICB were validated in the subsequent analysis. Based on an immunophenoscore developed by Charoentong P. et al. to predicting the response to immunotherapy [29], we found either anti-PD1 immunotherapy alone or anti-CTLA4 immunotherapy or the combination of anti-PD1 and anti-CTLA4 immunotherapy; the immunophenoscore was always higher in the low IRGscore group than in the high IRGscore group in the TCGA-SKCM cohort, which indicates that patients belonging to this group may benefit from these two types of immunotherapies (Fig. 5 a–c). In addition, after the analysis of data from the independent cohort consisting of CM patients receiving immunotherapy (GSE91061), it has been revealed that individuals with low IRGscores exhibited a prolonged survival than high score patients (Fig. 5d). Surprisingly, the further analysis indicated that in this cohort, all patients with clinical response, including partial response (PR)/complete response (CR), belonged to the low IRGscore group, suggesting IRGscores were extremely sensitive in predicting immunotherapeutic benefits (Fig. 5e). Collectively, these findings concluded that IRGscore was capable of serving as a therapeutic and prognostic biomarker, thereby assessing the immunotherapy response.

## Discussion

Immune checkpoint inhibitors targeting three distinct molecules (CTLA-4, PD-1 as well as its ligand PD-L1) have been approved by the US Food and Drug Administration for use in humans, improving the prognosis of patients with CM [5, 35]. Nevertheless, an obvious limitation restriction of ICB is merely a minor percentage of CM patients with durable responses could benefit from it, whereas the majority experienced little clinical benefit, far from meeting clinical needs [13]. Consequently, it is extremely necessary to determine appropriate individuals with CM for immunotherapy. And in our present work, we have constructed an effective approach, IRGscore, to quantify immune-related gene expression pattern and the comprehensive state of TIM in CM. In addition, this study further indicated that the IRGscore is a dependable predictor in evaluating the response to immunotherapy and an effective prognostic marker.

The TME comprises a variety of immune cells together with endothelial cells, fibroblasts, and extracellular components, having a profound impact on the tumor initiation, progression, and metastasis as well as the response to ICB [9]. The objective response to immune checkpoint blockade (ICB) therapy, aimed to promote efficacious anti-tumor immune responses, is based on the immune-related compositions of TME [36]. TILs, as predictors of response to ICB and regulator of tumor progression, have been widely applied to identify appropriate patients for immunotherapy [12, 37]. However, mounting evidence [13, 14] indicated that simply evaluating TIL cannot absolutely predict the response to immunotherapy, which was also impacted by various chemokines, cytokines, and other immune components of TME.

We believed the expression pattern of immune-related genes and the comprehensive characterization of the TIM could be the potential methodologies to predict the response of CM patients to immunotherapy and develop personalized treatment strategies. We identified a gene set related to immune via literature and survival analysis based on a cohort of CM samples was performed for each gene, obtaining 742 genes with prognostic value. Then, the CM patients, based on these genes, were classified into three distinct immune subtypes, showing significant differences in survival. Further analysis revealed the landscape of infiltrating immune cell of TIM. In immune cluster A with distinct survival advantage, immune-related signaling pathways were obviously activated, and the infiltrating levels of various immune cells, including activated B cell, CD8 T cell, NK cell, CD4 T cell, as well as activated DC cell, were dramatically higher than the others, suggesting the IRGscore and the level of infiltrating immune cells were significantly positively correlated to the individual’s survival. This finding was consistent with previous researches [12, 38].

Further analysis indicated that the differences in mRNA transcriptome among three subtypes were closely related to the immune-related biological pathways. Firstly, the DEGs were fetched among the three immune clusters, and next KEGG and GO enrichment analysis indicated that these DEGs were chiefly reinforced on biological procedures remarkably related to immune function, involving T-cell activation, leukocyte proliferation, lymphocyte differentiation, and mononuclear cell differentiation. These DEGs with prognosis value were deemed as immune signature genes. Consistent with the immune-related gene-based clustering analysis (immune clusters A, B, C), we discovered three genomic subtypes (gene lusters A, B, C) based on the selected immune signature genes. Further analysis demonstrated that gene cluster C had the lowest stromal score and immune score, as well as other cells related to immune response, suggesting an immunosuppressive phenotype. Interestingly, the infiltrating degrees of M2 macrophages have been observed in cluster C and were significantly higher compare to other subtypes, whereas the infiltrating degrees of M1 macrophages were the lowest. Previous studies [39, 40] indicated M2 macrophages could secrete many immunosuppressive cytokines, facilitating the progression and metastasis of tumors, which was associated with poor prognosis of tumors. M1 macrophages are generally considered to be tumor-killing macrophages, which mainly exert the anti-tumor effects. Conversely, we found the gene cluster A had prominently higher immune scores than other gene clusters, and it exhibited the highest activated CD8+ T cell and CD4+ T-cell infiltration, which played a central role in mediating responses to immunotherapy and controlling tumor growth. Additionally, as the targets for immunotherapy, the expression levels of several vital immune checkpoints were also investigated in three gene clusters. The gene cluster A was related to much higher expression levels of immune checkpoints, whereas gene cluster C revealed the lowest expression level. Numerous studies demonstrated that TIM had a crucial impact on the patient’s survival. Consistent with these studies, our results revealed that the immunosuppressive phenotype of gene cluster C was closely related to a poor prognosis which might contribute to immune evasion of tumor cells and produce resistance to immunotherapy, while the gene cluster A with strong immune response has a favorable prognosis. This further indicated the importance of comprehensively evaluating the patient’s immune status, which might help estimate the response to immunotherapy and prognosis.

Considering the individual heterogeneity of TIM, it was highly necessary to establish a scoring system to quantify immune status of individual tumors, assisting the formulation of individualized therapeutic strategies. Based on the above immune signature genes, our present work constructed a scoring pattern to quantify the immune function of individuals with CM, termed as IRGscore. Through GSEA, we found signaling pathways related to immune activation, such as NK cell-mediated cytotoxicity, B-cell receptor, T-cell receptor, and toll-like receptor signaling pathways, were significantly enriched in the low IRGscore group. The expression levels of immune checkpoint were also considered to influence an individual’s response to cancer immunotherapy. As expected, the expression levels of genes related to various immune checkpoint, including PD-1, PD-L1, and CTLA-4, exhibited dramatically higher in low score group, compared to the high score group, which further indicated the profound effect of IRGscore on evaluating the response to immunotherapy. Additionally, increasing researches [32, 41, 42] demonstrated tumor burden mutation (TMB) could be one of the key factors that determine a patient’s response to ICB; therefore, we further explore the correlation between the IRGscore and TMB. Nevertheless, the results revealed TMB had no prominent variations between the low and high IRGscores, and the prognostic values of immune score were independent of TMB in CM, suggesting this score system was an independent survival predictor.

The predictive value of IRGscore was further assessed based on the cohort (GSE91061) consisting of CM patients receiving immunotherapy, and the results indicated patients with low IRGscores exhibited a prolonged survival than high score patients. Furthermore, all patients with clinical response, including complete response (CR)/partial response (PR), belonged to the low IRGscore group, suggesting IRGscore was an extremely sensitive predictor of immunotherapeutic benefits. Additionally, based on an immunophenoscore to predict response to immunotherapy developed by Charoentong P. et al., we further demonstrated that CM patients with low IRGscore might benefit from anti-PD1 immunotherapy or anti-CTLA4 immunotherapy or the combination of anti-CTLA4 and anti-PD1 immunotherapy. Furthermore, the IRGscore and immune-related gene expression pattern were established through the use of retrospective datasets in this study. Thus, more prospective studies, including CM patients with immunotherapy, are required to examine our results. In addition, some patients with low IRGscore do not benefit obviously from immunotherapy, and other clinical or pathological characteristics are supposed to be incorporated into the predictive model to enhance the accuracy.

## Conclusion

To summarize, our present work revealed that characterization of TIM and the immune status of individuals were closely correlated to tumor heterogeneity and treatment complexity. A scoring system, IRGscore, was established to comprehensively evaluate immune-related gene expression pattern and the characterization of TIM of individuals with CM, providing a basis for determination of tumor immunophenotype and effective clinical practice. Furthermore, it has been demonstrated that this IRGscore system could serve as an independent prognostic and immunotherapeutic predictor, thereby facilitating evaluation of immunotherapy response in patients with CM and the identification of appropriate candidates for immunotherapy as well as the formulation of individualized therapeutic strategies.

## Supplementary Information

**Additional file 1: Supplementary Figure 1.** The workflow of this study and consensus matrixes of screened genes.

**Additional file 2: Supplementary Figure 2.** The correlation of IRGscore with tumor mutation burden.

## Data Availability

GSE65904, GSE91061, and GSE84437 datasets were preliminarily extracted from the National Center for Biotechnology Information (NCBI) Gene Expression Omnibus (GEO) repository (https://www.ncbi.nlm.nih.gov/geo/). Additional transcriptome RNA expression data (TCGA-SKCM and TCGA-STAD) were downloaded from TCGA (https://portal.gdc.cancer.gov/). All data generated or analyzed during this study are included in this published article.

## Abbreviations

CM: Cutaneous melanoma
TME: Tumor microenvironment
TIM: Tumor immune microenvironment
DEGs: Differentially expressed genes
GEO: Gene Expression Omnibus
KEGG: Kyoto Encyclopedia of Genes and Genomes
TCGA: The Cancer Genome Atlas
ICB: Immune checkpoint blockade
ssGSEA: Single-sample gene set enrichment analysis
GSVA: Gene set variation analysis
PCA: Principal component analysis
IPS: Immunophenoscore
