# Supplementary figures and images for: The expression pattern of immune-related genes and characterization of tumor immune microenvironment: predicting prognosis and immunotherapeutic effects in cutaneous melanoma
Source: World J Surg Oncol. 2022 Sep 22;20:303. doi: 10.1186/s12957-022-02767-z (PMC9502579; doi:10.1186/s12957-022-02767-z)

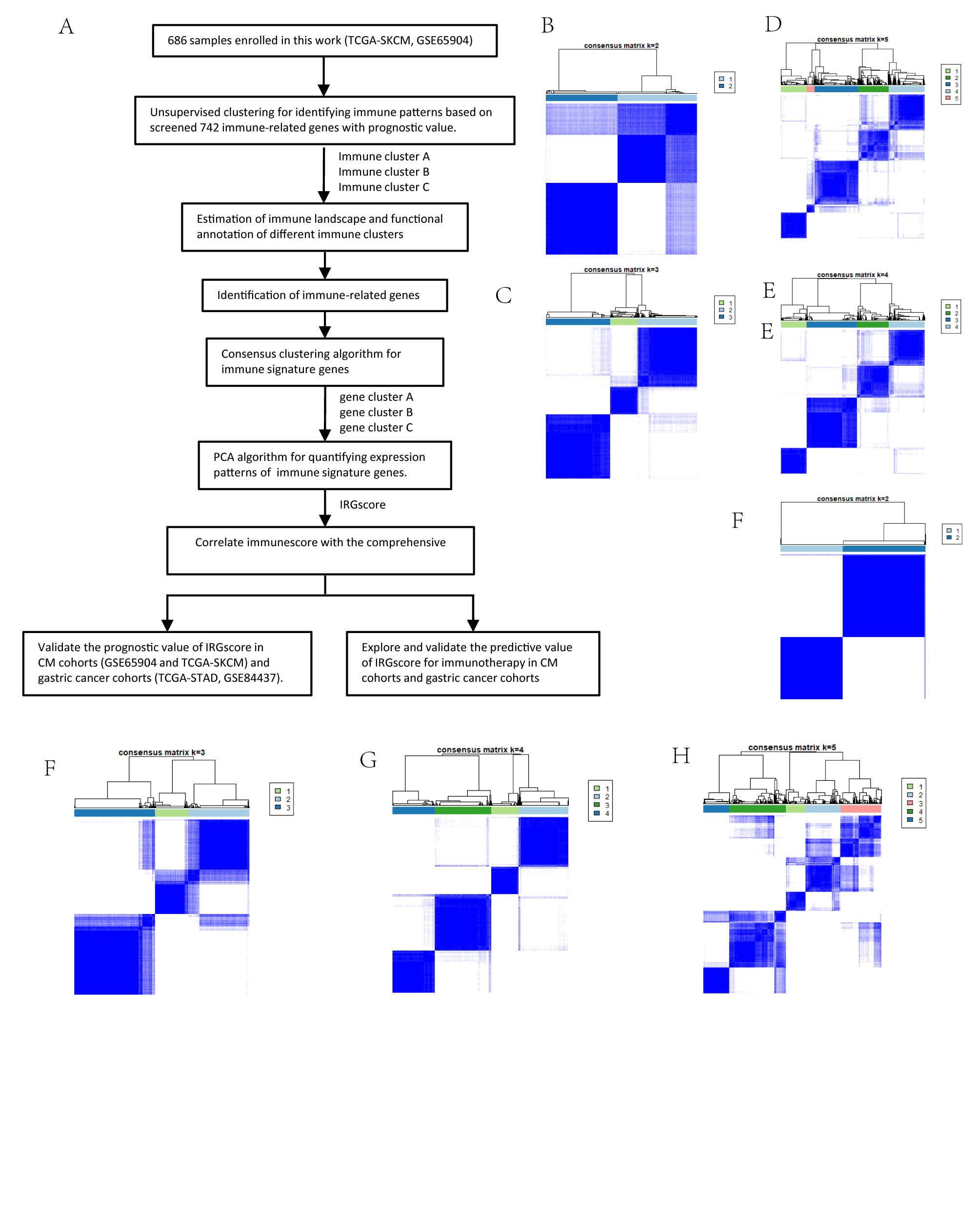

Supplement: Supplementary file 1 — Additional file 1: Supplementary Figure 1. The workflow of this study and consensus matrixes of screened genes. [file 12957_2022_2767_MOESM1_ESM.tif]

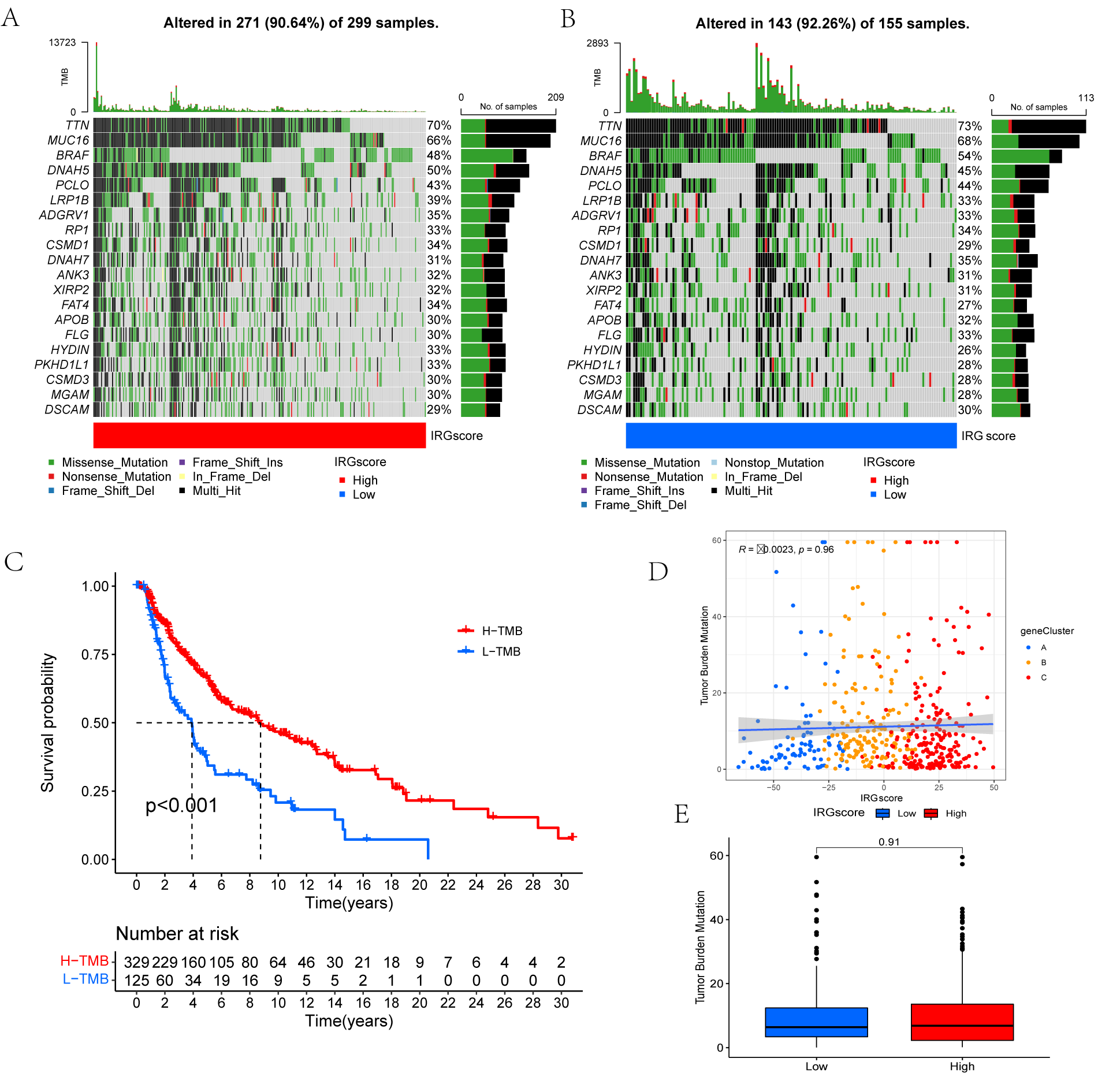

Supplement: Supplementary file 2 — Additional file 2: Supplementary Figure 2. The correlation of IRGscore with tumor mutation burden. [file 12957_2022_2767_MOESM2_ESM.tif]
